# Physicochemical Characterization, Antioxidant and Immunostimulatory Activities of Sulfated Polysaccharides Extracted from *Ascophyllum nodosum*

**DOI:** 10.3390/molecules23081912

**Published:** 2018-07-31

**Authors:** Ligen Chen, Yan Wang, Hui Yang, Han Li, Wei Xu, Guijie Chen, Hongjun Zhu

**Affiliations:** 1 College of Chemistry and Molecular Engineering, Nanjing Tech University, Nanjing 211816, China; chenlg@ycit.cn (L.C.); 1954357344@njtech.edu.cn (Y.W.); zllz@ycit.cn (H.L.); 2 School of Marine and Bioengineering, Yancheng Institute of Technology, Yancheng 224051, China; yangh96@yahoo.com; 3 Jiangsu Key Laboratory of Biochemistry and Biotechnology of Marine Wetland, Yancheng Institute of Technology, Yancheng 224051, China; 4 College of Food Science and Technology, Nanjing Agricultural University, Nanjing 210095, China; guijiechen@njau.edu.cn

**Keywords:** *Ascophyllum nodosum*, polysaccharides, antioxidant activity, immunostimulatory activity

## Abstract

Polysaccharides from *Ascophyllum nodosum* (AnPS) were extracted and purified via an optimized protocol. The optimal extraction conditions were as follows: extraction time of 4.3 h, extraction temperature of 84 °C and ratio (*v*/*w*, mL/g) of extraction solvent (water) to raw material of 27. The resulting yield was 9.15 ± 0.23% of crude AnPS. Two fractions, named AnP1-1 and AnP2-1 with molecular weights of 165.92 KDa and 370.68 KDa, were separated from the crude AnPS by chromatography in DEAE Sepharose Fast Flow and Sephacryl S-300, respectively. AnP1-1 was composed of mannose, ribose, glucuronic acid, glucose and fucose, and AnP2-1 was composed of mannose, glucuronic acid, galactose and fucose. AnPS, AnP1-1 and AnP2-1 exhibited high scavenging activities against ABTS radical and superoxide radical, and showed protective effect on H_2_O_2_-induced oxidative injury in RAW264.7 cells. Furthermore, the immunostimulatory activities of AnP1-1 and AnP2-1 were evaluated by Caco-2 cells, the results showed both AnP1-1 and AnP2-1 could significantly promote the production of immune reactive molecules such as interleukin (IL)-8, IL-1β, interferon (IFN)-γ, and tumor necrosis factor (TNF)-α. Therefore, the results suggest that AnPS and its two fractions may be explored as a potential functional food supplement.

## 1. Introduction

Edible marine macroalgae, or seaweeds, products have been traditionally used in Chinese herbal medicine for more than 600 years [1]. Marine macroalgae are commonly classified into three groups based on their pigmentation: brown (Phaeophyceae), red (Rhodophyceae) and green (Chlorophyceae) algae [2]. *Ascophyllum nodosum* is one of the most economically important brown algae in Britain, Peru and many other countries [3]. Compared with red and green algae, brown algae were reported to contain higher content of active components and showed stronger antioxidant activity [4]. The major components of brown algae extracts are polysaccharides, phenolic compounds, proteins, pigments, peptides, polyunsaturated fatty acids and terpenoids [5]. Recently, increasing attentions have been paid to brown algae polysaccharides due to their pharmacological activities, such as anticoagulant, antitumor, immunomodulatory, antiviral, antihyperlipidemic and antioxidant activity [6,7,8,9]. A growing body of evidences has demonstrated that the immunostimulatory activity is one of the most important biological activities of polysaccharides, which may serve as an important mechanism for its antitumor effect [10]. Intestine is an important immune organ of the human body. Non-starch polysaccharides can resist the digestion and hydrolyzation in the human saliva, stomach and small intestine [11], which thus reaches the intestine safely, where they may act as potential immune stimulator. Yu et al. [12] reported that EPS1-1, extracted from the fermentation broth of *Rhizopus nigricans*, was an extracellular polysaccharide, which play an important role in the improvement of intestinal function in mice with colorectal cancer. Kim et al. [13] reported that BLE-P, isolated from barley leaves, was a mixture of hemicellulosic glucuronoarabinoxylan and pectic rhamnogalacturonan. The results indicated that BLE-P not only augmented the production of immunoglobulin A (IgA), but also increased the levels of IgA-related cytokines, such as transforming growth factor-b and interleukin-10. Brito et al. [14] reported that PLS, extracted from the algae *Hypnea musciformis,* was sulfated polysaccharides, which reduced the colitis and all analyzed biochemical parameters. However, few reports were focus on the intestinal immunostimulatory activities of sulfated polysaccharides from *Ascophyllum nodosum* and the separation and characterization of polysaccharides from *Ascophyllum nodosum* [3]. To the best of our knowledge, this is the first attempt to investigate the antioxidant activities of different fractions from crude AnPS.

In this study, *Ascophyllum nodosum* was selected as the representative of brown algae, which was reported to contain much higher total polysaccharide than that of other bioactive compounds. The conditions required to extract polysaccharides from *Ascophyllum nodosum* were explored by means of response surface methodology (RSM) on the basis of a Box-Behnken design (BBD). The predicted conditions were used to purify the AnPS by DEAE Fast Flow cellulose anion-exchange column and Sephacryl S-300 size exclusion column. The obtained fractions, AnP1-1 and AnP2-1, were characterized by FT-IR and HPLC. The antioxidant activities of AnPS, AnP1-1 and AnP2-1 were evaluated in ABTS, DPPH radical scavenging, superoxide anion radical scavenging and reducing power assays. The protective effect on H_2_O_2_-induced oxidative injury in RAW264.7 cells was investigated. Furthermore, the intestinal immunostimulatory activities of the two fractions were also examined for the first time.

## 2. Results and Discussion

### 2.1. Optimization of AnPS Extraction Conditions

The experiment design for extraction yield is shown in Table 1, and the results were analyzed using Design-Expert 8.0.6 software. The relationship between the independent variables and the dependent variables was described by the following second-order polynomial equation [15]:

Y = 9.01 + 0.70X_1_ + 0.18X_2_ + 0.62X_3_ + 0.015X_1_X_3_ − 0.91X_1_^2^ − 0.33X_2_^2^ − 0.83X_3_^2^ (1)

where X_1_, X_2_ and X_3_ represents extraction temperature, extraction time and ratio of water to raw material, respectively.

ANOVA was used to determine significance and suitability in the model (Table 2). The result indicated that the *F*-value of the model is 49.98 with a low probability *p*-value (<0.0001), which implied that the model was significant. The values of both the determination coefficient (R^2^ = 0.9847) and the adjusted determination coefficient (adj-R^2^ = 0.9651) were high, which suggested good agreement between the experimental and the predicted values of the AnPS yield. The results suggested that the extraction temperature (X_1_) was the most significant parameter affecting the yield of AnPS, followed by extraction time (X_2_) and ratio of water to raw material (X_3_).

**Figure 1:**
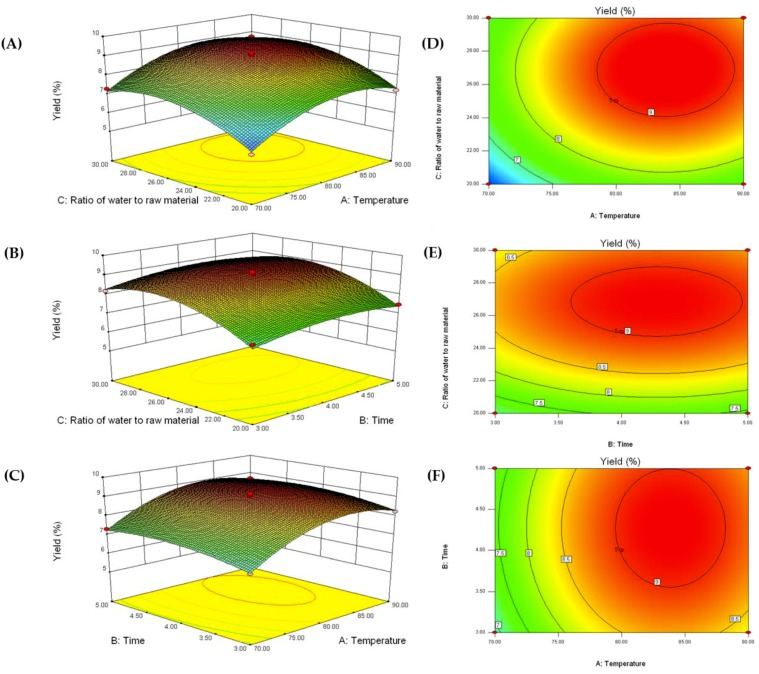
Response surface plots (**A**–**C**) and contour plots (**D**–**F**) showing the effects of two varied parameters (extraction temperature, extraction time, ratio of water to material) on the extraction yield of *Ascophyllum nodosum* polysaccharides (AnPS).

Figure 1A,D, depict the changes in extraction yield when the extraction temperature and extraction time varied across the experimental range when the ratio of water to raw material was fixed at zero. The yield is predicted to increase as the extraction temperature is elevated from 50 to 80 °C. At higher temperatures, the yield is predicted to slightly decrease. Generally, higher extraction temperature can promote the release of polysaccharides from plant tissues, but further increases in extraction temperature may induce polysaccharide degradation [16]. A similar trend was also predicted when extraction time and ratio of water to raw material were varied, as shown in Figure 1B,C.

According to the above RSM results, the optimal extraction conditions were obtained as follow: Extraction temperature of 83.9 °C, extraction time of 4.3 h, and a ratio of water to raw material of 26.9 mL/g. Under these conditions, the maximum predicted yield of AnPS was 9.3%. To validate the RSM model, a verification experiment was carried out under the optimized conditions, affording a AnPS yield of 9.2 ± 0.23% (n = 3). Notably, the yield of AnPS was higher than that of some polysaccharides (2.84–6.81%) from other brown seaweeds [17,18]. The result suggested that the conditioned modeled by RSM could be used in practice for the extraction of polysaccharides from *Ascophyllum nodosum*.

### 2.2. Isolation and Purification of Polysaccharide Fraction AnP1-1 and AnP2-1

**Figure 2:**
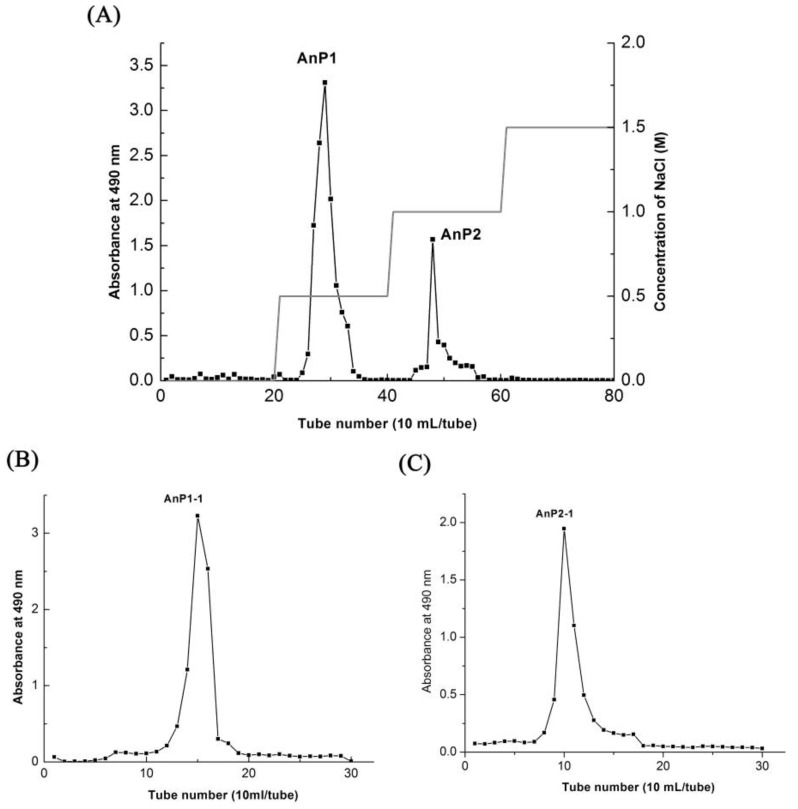
Stepwise elution curve of crude AnPS on DEAE Fast Flow column (**A**) and elution curves of polysaccharide fractions AnP1-1 (**B**), AnP2-1 (**C**) on Sephacryl S-300 column.

The crude AnPs was purified by DEAE Sepharose Fast Flow via a gradient elution of 0–1.5 M NaCl into two independent fractions (AnP1 and AnP2) as showed in Figure 2A. The peaks AnP1 and AnP2 were further purified by Sephacryl S-300 size exclusion chromatography using 0.1 M NaCl as the mobile phase (Figure 2B,C) to obtain the fractions of AnP1-1 and AnP2-1.

### 2.3. Characterization, Molecular Weight and Monosaccharide Composition of AnP1-1 and AnP2-1

The contents of carbohydrate, protein, total polyphenols, uronic acid and sulfate group in the AnPS, AnP1-1 and AnP2-1 fractions are shown in Table 3. The contents of carbohydrate in AnPS, AnP1-1 and AnP2-1 were 42.3%, 26.6% and 36.7%, respectively. The contents of protein in AnPS and its fractions were very low (1.4%, 0.5% and 0.4% for AnPS, AnP1-1 and AnP2-1, respectively). The total polyphenols content of crude AnPS (2.11 GAE/100 mg) was higher than that of AnP1-1 (0.13 GAE/100 mg) and AnP2-1 (0.11 GAE/100 mg), indicating that most of the polyphenols in AnPS was removed after the purification. The contents of uronic acid in AnPS, AnP1-1 and AnP2-1 were 11.0%, 13.5% and 3.6%, respectively. The contents of sulfate groups in AnPS, AnP1-1 and AnP2-1 were 23.9%, 5.4% and 23.8%, indicating that they should be sulfated polysaccharides.

The molecular weights of AnP1-1 and AnP2-1 were evaluated by HPGPC, and single and symmetrical peaks were detected, indicating that they are homogeneous polysaccharide. The average molecular weight of AnP1-1 was calculated to be 165.92 KDa and of AnP2-1 to be 370.68 KDa, according to the standard D-series dextrans.

The monosaccharide composition of AnPS, AnP1-1 and AnP2-1 were analyzed by HPLC (Table 3). AnPS was composed of Man, GlcA, Glc, Gal, Xyl and Fuc in the molar ratio of 1:1.22:0.10:0.58:0.21:2.90. AnP1-1 was composed of Man, Rib, GlcA, Glc and Fuc in the molar ratio of 1:0.40:0.45:0.32:3.44. AnP2-1 was mainly composed of Man, GlcA, Gal and Fuc in the molar ratio of 1:1.50:1.39:8.32. The results indicated that AnPS, AnP1-1 and AnP2-1 were heteropolysaccharides, and the fucose was the major monosaccharide.

### 2.4. FT-IR Analysis

**Figure 3:**
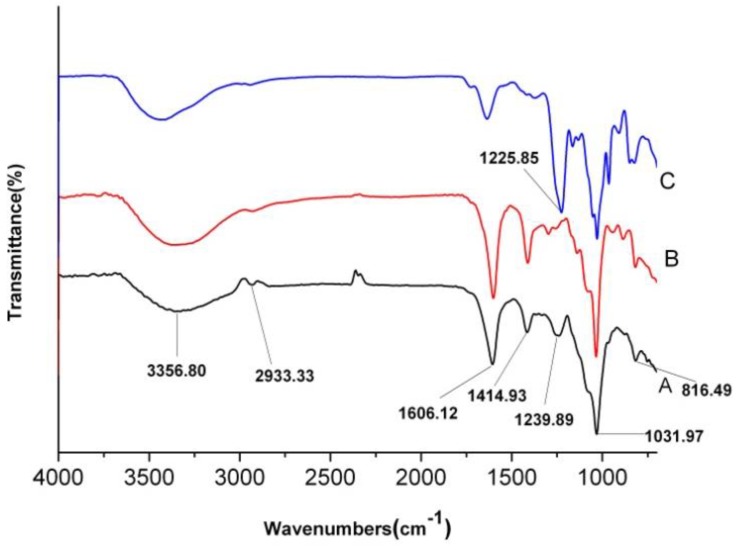
FT-IR spectra of AnPS (**A**), AnP1-1 (**B**) and AnP2-1 (**C**).

The FT-IR spectra of AnPS (A) and its purified fractions, AnP1-1 (B) and AnP2-1 (C), are shown in Figure 3. The broad and strong absorption peak around 3400 cm^−1^ could be attributed to O-H stretching vibrations, and a weak peak at about 2900 cm^−1^ could be attributed to C-H stretching vibrations. The band of at 1606 cm^−1^ and 1414 cm^−1^ was assigned to the COO- antisymmetrical stretching band and C=O symmetric stretching vibration of the carboxylate group of uronic acid.

Compared with C, the band at 1606 cm^−1^ and 1414 cm^−1^ was much higher in A and B, indicating that the uronic acid in C was much lower than A and B. The absorption at 1225–1240 cm^−1^ (S=O stretching vibration) confirmed the presence of sulfates in A and C. The band at 1239 cm^−1^ in C was much higher than that in A, indicating that the sulfate group was much higher than C. No band at 1225–1240 cm^−1^ was found in B, indicating that there was little sulfate group in B [19]. The band at 816 cm^−1^ suggested A and B were mainly sulfated at C-2 and/or C-3 on fucose or C-2 on galactose residues. However, the band of C-O-S vibration of C shifted to 844.6 cm^−1^, indicating the sulfation at C-4 on fucose residues [20].

### 2.5. Antioxidant Activity of A. nodosum Polysaccharides

**Figure 4:**
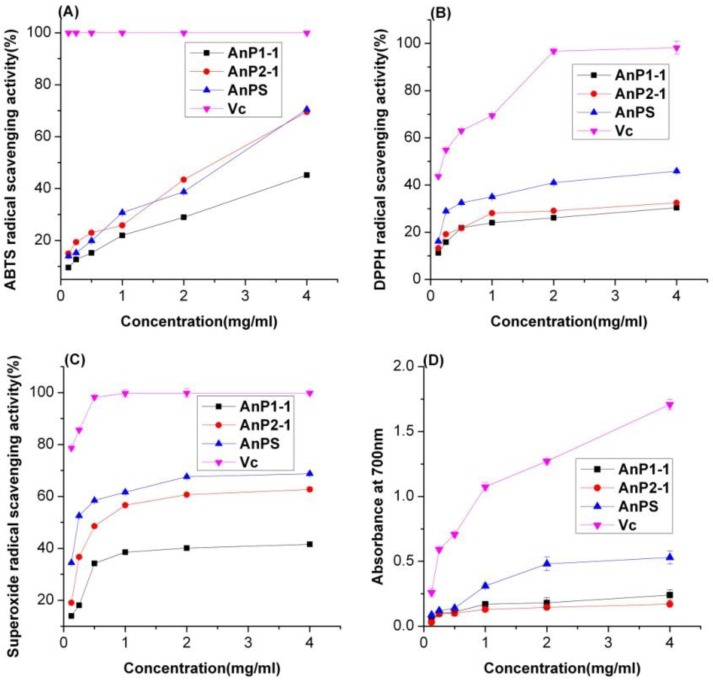
Scavenging abilities of *A. nodosum* polysaccharides from crude extract (AnPS) and two purified fractions (AnP1-1 and AnP2-1) on ABTS radical (**A**), DPPH radical (**B**) and superoxide radical (**C**) and their reducing power (**D**).

As shown in Figure 4A, all the samples showed increased scavenging activity in a concentration-dependent manner. At the polysaccharide concentration of 4 mg/mL, the AnPS, AnP1-1 and AnP2-1 extracts scavenged 70.5%, 45.2% and 69.5%, respectively, of the ABTS radical, while the positive control Vc scavenged 100%. This shown that the AnPS and its purified fractions had strong ABTS scavenging activity. AnP2-1 displayed superior ABTS radical scavenging activity, which might be due to the higher Fuc content (Table 3) [21].

The scavenging effects on DPPH free radicals were presented in Figure 4B. The scavenging activity of the polysaccharides increased with an increase in their concentration. At a concentration of 4 mg/mL, the DPPH free radical scavenging activities of crude AnPS, AnP1-1, AnP2-1 and Vc were 45.9%, 30.4%, 32.5% and 98.2%, respectively. Compared with the results of the ABTS assay, the AnPS and its purified fractions had limited scavenging abilities for DPPH. The reason might be due to the facts that the ABTS assay is more suitable for evaluating hydrophilic antioxidants [22].

The superoxide radical scavenging activity of AnPS and its purified fractions were measured by the PMS/NADH-NBT system (Figure 4C). For all samples, the superoxide anion radical scavenging activities increased with increasing sample concentrations, which was similar to those of the ABTS and DPPH assays. At the concentration of 4.0 mg/mL, the superoxide anion radical scavenging activities of AnPS, AnP1-1, AnP2-1 and Vc were 68.8%, 41.6%, 62.7% and 99.8%, respectively. The AnPS and AnP2-1 showed stronger scavenging activity than AnP1-1. This may partly be related to the higher sulfate group content in AnPS and AnP2-1 [23]. As shown in Figure 4D, the reducing power of the AnPS and its purified fractions increased with increasing concentration. Compared with Vc, the AnPS, AnP1-1 and AnP2-1 fractions exhibited significantly lower reducing power. At a concentration of 4.0 mg/mL, the reducing power was 0.53, 0.24, 0.17 and 1.71 for AnPS, AnP1-1, AnP2-1 and Vc, respectively, and the reducing power was 30%, 12% and 10% of Vc. The AnPS showed stronger reducing power than the AnP1-1 and AnP2-1, which might partly be due to the ratio of sulfate/fucose [24].

### 2.6. Protective Effect on H_2_O_2_-Induced Oxidative Injury

**Figure 5:**
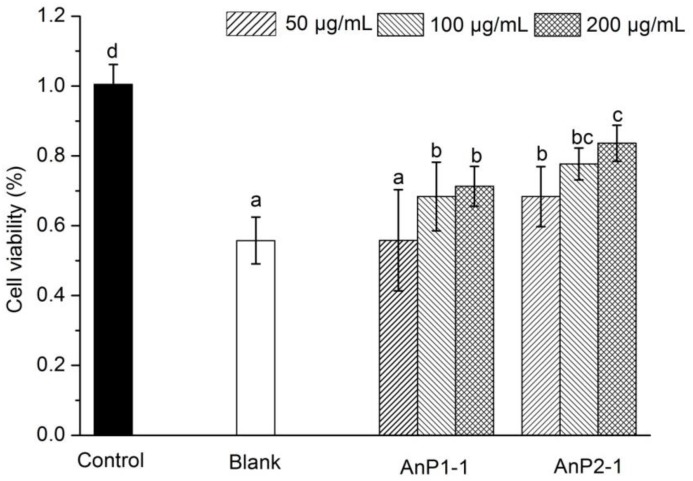
The protective effect of AnP1-1 and AnP2-1 against H_2_O_2_-induced oxidative injury in RAW264.7 cells. The different letters represent significant differences between different groups (*p* < 0.05).

H_2_O_2_ was considered as a by-product of dopamine oxidation and enzymatic action, which may lead to neuronal damage and oxidative stress through release of reactive oxygen species (ROS) [24]. Furthermore, large numbers of reports shows that oxidative injury is related to a wide range of disease, including diabetes mellitus, arteriosclerosis, Alzheimer’s disease, nephritis and even cancer, due to the excessive production of ROS [10]. In this work, the protective effect of AnP1-1 and AnP2-1 on H_2_O_2_-induced oxidative injury was carried out to verify the antioxidant in cellular level. As showed in Figure 5, H_2_O_2_ solution treatment significantly induced oxidative injury in RAW264.7 cells, and the cell viability decreased from 100.0 ± 5.7% to 55.8 ± 6.7%, suggested that the model was reliable. As expected, the pre-incubation with AnP1-1 and AnP2-1 could significantly increase the cell viability in a dose-dependent manner, indicated that AnP1-1 and AnP2-1 could attenuate the H_2_O_2_-induced oxidative injury in RAW264.7 cells. Notably, AnP2-1 showed higher protective effect on RAW264.7 cells than AnP1-1, which might be due to the higher content of sulfate group in AnP2-1. Likewise, Di et al. also found that polysaccharides from *Gracilaria rubra* with higher content of sulfate groups had stronger antioxidant activities [25].

### 2.7. Immunostimulatory Activity on Caco-2 Cells

**Figure 6:**
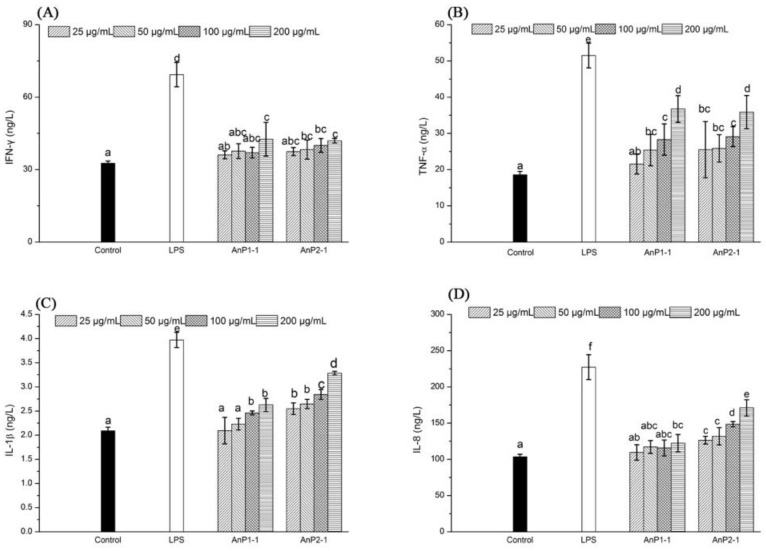
Effects of AnP1-1 and AnP2-1 on Cytokine IFN-γ (**A**), TNF-α (**B**), I L-1β (**C**) and IL-8 (**D**) Levels of Caco-2 Cells. The different letters represent significant differences between different groups (*p* < 0.05).

The cytokines, which are small molecule proteins produced by immune cells, could reflect the abilities of immunomodulatory and anti-inflammatory [26]. In the present study, the effects of AnP1-1 and AnP2-1 under different concentrations (0, 25, 50, 100 and 200 μg/mL) on the production of cytokines, including IFN-γ, TNF-α, IL-1β, and IL-8, were investigated in the Caco-2 cell model. The results showed that both AnP1-1 and AnP2-1 had limited effect on the levels of IFN-γ (Figure 6A). As expected, the minimum levels of TNF-α, IL-8, and IL-1β were observed in untreated Caco-2 cells, where as the productions of TNF-α, IL-8, and IL-1β were significantly enhanced after the treatment of AnP1-1. The AnP2-1 treatment could also facilitate the release of TNF-α, IL-8, and IL-1β in a dose-dependent manner, which showed much better than that of AnP1-1. It has been reported that a heteropolysaccharide L2 from *Lentinula edodes* could also enhance the production of anti-inflammatory cytokine IL-10 and proinflammatory cytokines (IFN-γ, TNF-α, IL-8 and IL-12) in Caco-2 cells [27]. The results indicated that both AnP1-1 and AnP2-1 could stimulate immune responses of Caco-2 cells through the release of immune cell factors. Recently, there is growing number of work has demonstrated that sulfated polysaccharides, especially from brown and green seaweeds, had superior immunomodulatory activities, which was related to the high contents of sulfate groups [25]. In the present study, the contents of sulfate groups in AnP1-1 and AnP2-1 were 5.4 ± 0.11 and 23.8 ± 0.02, respectively, which may result in a much stronger immunostimulatory activity of AnP2-1. Unfortunately, the potential molecular mechanism of immunostimulatory activity of AnP1-1 and AnP2-1 is still unknown, it will be our next work.

## 3. Materials and Methods

### 3.1. Materials and Chemicals

The sample of *Ascophyllum nodosum* was obtained from Mingyue Seaweed Corporation (Qindao, Shangdong, China). DEAE Sepharose Fast Flow and Sephacryl S-300 were purchased from GE (Uppsala, Sweden). 1-phenyl-3-methyl-5-pyrazolone (PMP), D-series dextran standards and standard monosaccharides were purchased from Sigma-Aldrich Co., Ltd. (St. Louis, MO, USA). Nitrotetrazolium blue chloride (NBT), phenazine methosulfate (PMS), 2,2’-azinobis-(3-ethylbenzoth-iazoline-6-sulfonic acid) (ABTS) and 2,2-diphenyl-1-picrylhydrazyl (DPPH) were purchased from Aladdin Chemical Reagent Co., Ltd. (Shanghai, China). Mulbecco’s modified Eagle medium (DMEM), fetal calf serum, trypsin-EDTA, streptomycin and penicillin were purchased from Gibco/Invitrogen (Carlsbad, CA, USA). The 3-(4,5-dimethylthiazol-2-yl)-2,5-diphenyltetrazolium bromide (MTT) and lipopolysaccharides (LPS) were obtained from Sigma Chemical Co. The RAW264.7 cells and Caco-2 cells were purchased from Keygen Co. (Nanjing, China). Enzyme-linked immunosorbent assay (ELISA) kits for IFN-γ, IL-8, TNF-α and I L-1β were purchased from Nanjing Jiancheng Bioengineering Institute (Nanjing, China). All other reagents and solvents were of analytical grade.

### 3.2. Extraction of Crude AnPS

The polysaccharides were extracted according to the previous method [28]. The dried *Ascophyllum nodosum* powder was extracted with 80% ethanol three times for 36 h in order to remove the small molecules, and then was extracted using hot water under the conditions determined by response surface methodology (RSM) of the designed extraction temperature, extraction time and ratio of water to raw material. The extract was combined, centrifuged and concentrated. The solution was deproteinated using the Sevag method [28], dialyzed and lyophilized to obtain the crude *Ascophyllum nodosum* polysaccharides (AnPS). The extraction yield was calculated according to the following formula:

Extraction yield (%) = W_1_/W × 100 (2)

where W_1_ and W are the weights of AnPS and the dried powder sample, respectively.

### 3.3. Design of Experimental (DoE) in Response Surface Methodology (RSM)

According to single-factor tests, a BBD with three independent variables (X_1_, extraction temperature; X_2_, extraction time; X_3_, ratio of water to raw material) at three levels was performed. The whole design consisted of 17 computed experimental runs (12 factorial point and 5 axial points). All the design points were modeled in triplicate in a randomized order. The experimental data (Table 1) were analyzed by multiple regressions to fit the following second-order polynomial mode [16]:

(3) $$Y=\beta_{0}+\sum_{i=1}^{3}\beta_{i}X_{i}+\sum_{i=1}^{3}\beta_{ii}X_{i}^{2}+\sum_{i=1}^{2}\sum_{j=i+1}^{3}\beta_{ii}X_{i}X_{j}$$

where Y is the predicted response (extraction yield of AnPS); *β*_0_, *β_i_*, *β_ii_* and *β_ij_* are the regression coefficients for intercept, linear, quadratic and interaction terms, respectively; X*_i_* and X*_j_* are the independent variables (*i* ≠ *j*) [15].

### 3.4. Purification of A. nodosum Polysaccharides (AnPS)

Crude AnPS was dissolved in deionized water (20 mL, 15 mg/mL) and loaded into a DEAE Sepharose Fast Flow chromatography column (2.6 cm i.d. × 60 cm), the column was stepwise eluted by 0, 0.5 and 1.0 M sodium chloride (NaCl) solution at a flow rate of 2.5 mL/min and the eluent was collected 10 mL per tube. Two completely separated fractions, AnP1 and AnP2, were collected by checking the absorbance at 490 nm by using phenol-sulfuric acid method [9]. Then, the polysaccharide fractions were dialyzed and purified further by Sephacryl S-300 size-exclusiong chromatography column (1.6 cm i.d. × 100 cm). The column was eluted with 0.1 M aqueous NaCl solution at a rate of 40 mL/h. As a result, the main fractions were collected, dialyzed and lyophilized, respectively, affording AnP1-1 and AnP2-1.

### 3.5. Analysis and Characterization of the Polysaccharide Fractions

The contents of carbohydrate in AnPS, AnP1-1 and AnP2-1 were measured by the phenol-sulphuric acid method [29]. The content of protein was determined according to the method of Bradford [30]. The content of total polyphenols was estimated by the Folin–Ciocalteu colori-metric method [31]. The content of uronic acid was estimated according to the method of Blumenkrantz and Asboe-Hansen [32]. The content of sulfate group was determined according to the reported method of Doigson and Price [33].

The homogeneity and molecular weight of AnP1-1 and AnP2-1 were determined using high performance gel permeation chromatography (HPGPC, Waters Technologies) equipped with a TSK-G 3000 PWxL column. The column temperature was set at 30 °C. The column was eluted with double distilled water at a rate of 0.7 mL/min. The D-series dextran standards (D-2, D-3, D-4, D-5, D-6, D-7 and D-8) were employed to obtain the calibration curve. The sample concentration was set at 2.0 mg/mL.

The monosaccharide composition of AnP1-1 and AnP2-1 were analyzed by HPLC using reported methods [33]. The sample (5 mg) was hydrolyzed with trifluoroacetic acid (TFA, 2 M) for 2 h at 120 °C. The resultant solution was cooled to the room temperature and was evaporated to dryness under reduced pressure repeatedly. The residue was dissolved in methanol (200 μL) and evaporated again to dryness. The residue was derived by PMP, according to the reported method [28]. The products were analyzed on a HPLC system (Agilent 1200) equipped with Eclipse Plus C18 column (4.6 × 250 mm, 5 μm, Agilent) and photodiode array detector. The chromatographic conditions were as follows: detector wavelength, 245 nm; column temperature, 30 °C; flow rate, 1.0 mL/min; mobile phase, a mixture of acetonitrile and phosphate buffered saline (PBS, 0.1 M, pH 6.7) in a ratio of 83:17 (*v*/*v*).

Dried polysaccharide samples (crude AnPS, AnP1-1 and AnP2-1) were mixed with KBr powder and the mixture was pressed into a pellet. The samles were characterized by FT-IR on a spectrophotometer (Nexus 6700, Bruker Co., Karlsruhe, Germany) in the range of 400–4000 cm^−1^.

### 3.6. Antioxidant Activity Assays

#### 3.6.1. ABTS Assay

The crude AnPS and the purified fractions AnP1-1 and AnP2-1 were subjected to an ABTS assay [15,34] with some modifications. The ABTS radical solution was generated through the reaction of potassium persulfate (3.8 mM) and ABTS (7.4 mM) for 16 h at 25 °C in the dark. The solution was diluted with PBS buffer (pH 7.4) to obtain an absorbance of 0.70 ± 0.01 at 734 nm. Ascorbic acid (vitamin C, Vc) was used as a positive standard. Sample solutions (Vc, AnPS, AnP1-1 and AnP2-1) were diluted to 0.125, 0.25, 0.5, 1.0, 2.0 and 4.0 mg/mL. The diluted samples (25 μL) were mixed with 250 μL ABTS solution. After mixing for 6 min at room temperature, the absorbance of the mixture was measured at 734 nm. The ABTS radical scavenging activity was calculated by the following formula:

ABTS radical scavenging activity (%) = [1 − (A_1_ − A_2_)/A_0_] × 100 (4)

where A_1_ is the absorbance of the sample solution, A_2_ is the absorbance of the solution containing sample and PBS, and A_0_ is the absorbance of the solution containing ABTS and distilled water.

#### 3.6.2. DPPH Radical Scavenging Activity

DPPH radical scavenging activity was determined using the reported method [35]. Briefly, 50 μL sample solution of different concentrations (0.125, 0.25, 0.5, 1.0 and 2.0 mg/mL) was added to 50 μL (0.2 mM) DPPH-methanol solution in a 96-well plate. After vigorous shaking, the reaction was kept in the dark at room temperature for 30 min. The absorbance was measured at 517 nm with Vc as a positive standard. The scavenging activity of DPPH radical was calculated by the following formula:

DPPH radical scavenging activity (%) = [1 − (A_1_ − A_2_)/A_0_] × 100 (5)

where A_1_ is the absorbance of the reaction solution, A_2_ is the absorbance of the solution containing sample and ethanol, and A_0_ is the absorbance of the solution containing DPPH and distilled water.

#### 3.6.3. Superoxide Anion Radical Scavenging Activity

The superoxide anion radical scavenging activity was measured according to the reported method [36] with minor modifications. In this experiment, 50 μL NADH solution (78 μM), 50 μL PMS solution (10 μM), 50 μL NBT solution (78 μM) and 50 μL sample solution (0.125, 0.25, 0.5, 1.0, 2.0 and 4.0 mg/mL) were mixed in a 96-well plate. The reaction solution was incubated at 25 °C for 5 min. The absorbance of the reaction was measured at 560 nm with V_C_ as positive control. The superoxide anion radical scavenging activity was calculated using the following formula:

Superoxide anion radical scavenging activity (%) = [1 − (A_1_ − A_2_)/A_0_] × 100 (6)

where A_1_ is the absorbance of the sample solution, A_2_ is the absorbance of the sample only (PBS buffer), and A_0_ is the absorbance of the control (distilled water).

#### 3.6.4. Reducing Power Assay

The reducing power of AnPS was quantified by the reported method with some modifications [37]. Briefly, 50 μL PBS (pH 6.6, 0.2 M), 50 μL potassium ferricyanide (1%, *w*/*v*) and 50 μL sample solutions (0.125, 0.25, 0.5, 1.0, 2.0 and 4.0 mg/mL) were mixed and incubated at 50 °C for 20 min in a 96-well plate. After cooling rapidly, 50 μL TCA solution (10%, *w*/*v*) was added to the mixture and centrifuged at 8000 rpm for 10 min. The supernatant solution was transferred and mixed with 25 μL ferric chloride (0.1%, *w*/*v*) and 25 μL of distilled water. The absorbance of the reaction mixture was recorded at 700 nm with Vc as positive control. The reducing power was calculated according to the formula:

Reducing power = A_1_ − A_2_ (7)

where A_1_ is the absorbance of reaction mixture of sample, A_2_ is the absorbance of sample solution without ferric chloride.

### 3.7. Protective Effect on H_2_O_2_-Induced Oxidative Injury

The protective effect of AnPS, AnP1-1 and AnP2-1 on H_2_O_2_-induced oxidative injury on RAW264.7 was carried out according to the previous method with some modification by MTT assay [25]. The RAW264.7 cells were cultured in DMEM supplemented with fetal calf serum (10%, *v*/*v*) and penicillin-streptomycin (1%, *v*/*v*) in a humidified atmosphere containing CO_2_ (5%) at 37 °C. 200 μL/well of RAW264.7 cells suspension was pipetted into a 96-well culture plate and incubated for 12 h. The culture medium was replaced by new culture medium with different concentrations (0, 50, 100 and 200 μg/mL) of samples (AnP1-1 and AnP2-1). After culture of 24 h, the culture medium with 850 μM of H_2_O_2_ was added after removal of the old culture medium and incubated for a further 2 h. Then, the medium was removed, 50 μL of MTT solution (2 mg/mL) was added and incubated for 4 h at 37 °C. Finally, 150 μL of DMSO was added into each well to replace MTT solution. The absorbance at 570 nm was measured with a microplate reader. The cell viability was calculated by the following equation:

Cell viability = As/A_0_ (8)

where As is the absorbance of the sample and A_0_ is the absorbance of blank.

### 3.8. Assay of Immunostimulatory Activity In Vitro

The assay of immunostimulatory activity in vitro was carried out using Caco-2 cells model according to the previous method [26]. 200 μL/well of Caco-2 cells suspension was pipetted into a 96-well culture plate and incubated for 12 h. The culture medium was replaced by new culture medium with different concentrations (0, 25, 50, 100 and 200 μg/mL) of samples (AnP1-1 and AnP2-1) or LPS (1 μg/mL). After culture of 24 h, the levels of IFN-γ, TNF-α, IL-1β, and IL-8 in the supernatants of the Caco-2 cells from each group were evaluated by using ELISA kits according to the manufacturers’ protocols (Nanjing Jiancheng Bioengineering Institute, Nanjing, China). LPS was used as positive control.

### 3.9. Statistical Analysis

All measurements were performed in triplicate and the average values were reported. Analysis of variance (ANOVA) followed by Duncan’s multiple-range tests with *P* < 0.05 were used to determine significant differences.

## 4. Conclusions

In this work, the optimized conditions for extraction of sulfated polysaccharide from *Ascophyllum nodosum* were determined as follows: extraction temperature of 84 °C, extraction time of 4.3 h and a ratio of water to raw material of 27 mL/g. Under these predicted conditions the polysaccharide yield was 9.2 ± 0.23%. Two fractions, AnP1-1 and AnP2-1, with molecular weights of 165.92 KDa and 370.68 KDa, respectively, were obtained by using chromatography with DEAE Sepharose Fast Flow and Sephacryl S-300. The monosaccharide composition of AnPS was composed of mannose, glucuronic acid, glucose, galactose, xylose and fucose, AnP1-1 was composed of mannose, ribose, glucuronic acid, glucose and fucose and AnP2-1 was composed of mannose, glucuronic acid, galactose and fucose. Crude AnPS, AnP1-1 and AnP2-1 exhibited high scavenging activities against ABTS and superoxide radicals, and protective effect on H_2_O_2_-induced oxidative injury in RAW264.7 cells. Furthermore, both AnP1-1 and AnP2-1 had stronger intestinal immunostimulatory activities evaluated by Caco-2 model. These results indicated that AnPS and its purified fractions could be used as potential antioxidants and intestinal immune stimulators. Further study is required to give more insight into the relationship between the mechanisms behind these biologically important activities and the structure of AnPS and its purified fractions.

## Figures and Tables

**Table 1:** Box-Behnken design matrix and the response values for the extraction yield of AnPS.

Run	Temperature (°C)		Time (h)		Ratio of Water to Raw Material (mL/g)		Predicted Value (%)	Experimental Value (%)
	X_1_	Code X_1_	X_2_	Code X_2_	X_3_	Code X_3_		
**1**	80	0	4	0	25	0	9.01	9.09
**2**	90	1	4	0	20	−1	7.34	7.22
**3**	70	−1	5	1	25	0	7.26	7.32
**4**	70	−1	3	−1	25	0	6.89	6.85
**5**	80	0	4	0	25	0	9.01	8.90
**6**	80	0	5	1	30	1	8.65	8.47
**7**	70	−1	4	0	30	1	7.17	7.29
**8**	80	0	3	−1	30	1	8.28	8.20
**9**	80	0	3	−1	20	−1	7.05	7.23
**10**	90	1	4	0	30	1	8.61	8.75
**11**	70	−1	4	0	20	−1	5.96	5.82
**12**	80	0	4	0	25	0	9.01	8.85
**13**	80	0	4	0	25	0	9.01	9.04
**14**	80	0	4	0	25	0	9.01	9.17
**15**	80	0	5	1	20	−1	7.41	7.49
**16**	90	1	5	1	25	0	8.65	8.69
**17**	90	1	3	−1	25	0	8.29	8.23

**Table 2:** ANOVA for response surface quadratic model of AnPS extraction.

Source	Sum of Squares	DF	Mean Square	*F*-Value	*p*-Value
Model	14.77	9	1.64	49.98	<0.0001
X_1_	3.93	1	3.93	119.77	<0.0001
X_2_	0.27	1	0.27	8.11	0.0248
X_3_	3.06	1	3.06	93.25	<0.0001
X_1_X_2_	0	1	0	0	0.9788
X_1_X_3_	0	1	0	0.027	0.8732
X_2_X_3_	0	1	0	0	0.9788
X_1_^2^	3.47	1	3.47	105.57	<0.0001
X_2_^2^	0.46	1	0.46	13.96	0.0073
X_3_^2^	2.92	1	2.92	88.84	<0.0001
Residual	0.23	7	0.033		
Lack of fit	0.16	3	0.053	3.01	0.1575
Pure error	0.071	4	0.018		
Cor. total	15	16			

**Table 3:** Preliminary characterization of AnPS, AnP1-1 and AnP2-1.

Item	AnPS	AnP1-1	AnP2-1
Carbohydrate (%)	42.31 ± 1.12	26.55 ± 1.12	36.69 ± 0.03
Protein (%)	1.43 ± 0.02	0.53 ± 0.01	0.42 ± 0.04
Total polyphenols (mg GAE/100 mg)	2.11 ± 0.04	0.13 ± 0.01	0.11 ± 0.01
Uronic acid (%)	11.04 ± 0.23	13.53 ± 0.79	3.56 ± 0.80
sulfate group (%)	23.9 ± 0.07	5.4 ± 0.11	23.8 ± 0.02
Molecular weight (kDa)	-	165.92	370.68
Monosaccharide composition (mol)			
Man	1	1	1
Rib	-	0.40	-
GlcA	1.22	0.45	1.50
Glc	0.10	0.32	-
Gal	0.58	-	1.39
Xyl	0.21		
Fuc	2.90	3.44	8.32

-: Not detected.

## References

[1] Chan C.X., Ho C.L., Phang S.M. (2006). Trends in seaweed research. Trends Plant Sci..

[2] Alamoudi O.A., Mutawie H.H., Patel A.V., Blunden G. (2009). Chemical composition and antioxidant activities of Jeddah corniche algae, Saudi Arabia. Saudi J. Biol. Sci..

[3] Yuan Y., Macquarrie D.C. (2015). Microwave assisted extraction of sulfated polysaccharides (fucoidan) from *Ascophyllum nodosum* and its antioxidant activity. Carbohydr. Polym..

[4] Kindleysides S., Quek S.Y., Miller M.R. (2012). Inhibition of fish oil oxidation and the radical scavenging activity of New Zealand seaweed extracts. Food Chem..

[5] Balboa E.M., Conde E., Moure A., Falqué E., Domínguez H. (2013). In vitro antioxidant properties of crude extracts and compounds from brown algae. Food Chem..

[6] Zhang Z.S., Wang F., Wang X.M., Liu X.L., Hou Y., Zhang Q.B. (2010). Extraction of the polysaccharides from five algae and their potential antioxidant activity in vitro. Carbohydr. Polym..

[7] Yang W.N., Chen P.W., Huang C.Y. (2017). Compositional characteristics and in vitro evaluations of antioxidant and neuroprotective properties of crude extracts of fucoidan prepared from compressional puffing-pretreated *Sargassum crassifolium*. Mar. Drugs.

[8] Yu P., Sun H. (2014). Purification of a fucoidan from kelp polysaccharide and its inhibitory kinetics for tyrosinase. Carbohydr. Polym..

[9] Han Y., Wu J., Liu T.T., Hu Y.D., Zheng Q.S., Wang B.S., Lin H.Y., Li X. (2016). Separation, characterization and anticancer activities of a sulfated polysaccharide from *Undaria pinnatifida*. Int. J. Biol. Macromol..

[10] Chen G.J., Yuan Q.X., Saeeduddin M., Ou S., Zeng X.X., Ye H. (2016). Recent advances in tea polysaccharides: Extraction, purification, physicochemical characterization and bioactivities. Carbohydr. Polym..

[11] Chen G.J., Xie M.H., Wan P., Chen D., Ye H., Chen L.G., Zeng X.X., Liu Z.H. (2018). Digestion under saliva, simulated gastric and small intestinal conditions and fermentation in vitro by human intestinal microbiota of polysaccharides from Fuzhuan brick tea. Food Chem..

[12] Yu Z.D., Song G., Liu J., Wang J.Y., Zhang P.Y., Chen K.S. (2018). Beneficial effects of extracellular polysaccharide from Rhizopus nigricans on the intestinal immunity of colorectal cancer mice. Int. J. Biol. Macromol..

[13] Kim H., Yu K.W., Hong H.D., Shin K.S. (2017). Effect of arabinoxylan- and rhamnogalacturonan I-rich polysaccharides isolated from young barley leaf on intestinal immunostimulatory activity. Funct. Foods.

[14] Brito T.V., Barros F.C.N., Silva R.O., Dias Júnior G.J., Júnior J.S.C., Franco Á.X., Soares P.M.G., Chaves L.S., Abreu C.M.W.S., de Paula R.C.M. (2016). Sulfated polysaccharide from the marine algae *Hypnea musciformis* inhibits TNBS-induced intestinal damage in rats. Carbohydr. Polym..

[15] Hammi K.M., Hammami M., Rihouey C., Cerf D.L., Ksouri R., Majdoub H. (2016). Optimization extraction of polysaccharide from Tunisian Zizyphus lotus fruit by response surface methodology: Composition and antioxidant activity. Food Chem..

[16] Samavati V., Yarmand M.S. (2013). Statistical modeling of process parameters for the recovery of polysaccharide from *Morus alba* leaf. Carbohydr. Polym..

[17] Ren B.B., Chen C., Li C., Fu X., You L.J., Liu R.H. (2017). Optimization of microwave-assisted extraction of *Sargassum thunbergii* polysaccharides and its antioxidant and hypoglycemic activities. Carbohydr. Polym..

[18] Cao C.L., Huang Q., Zhang B., Li C., Fu X. (2018). Physicochemical characterization and in vitro hypoglycemic activities of polysaccharides from *Sargassum pallidum* by microwave-assisted aqueous two-phase extraction. Int. J. Biol. Macromol..

[19] Thambiraj S.R., Phillips M., Koyyalamudi S.R., Reddy N. (2015). Antioxidant activities and characterisation of polysaccharides isolated from the seeds of *Lupinus angustifolius*. Ind. Crop. Prod..

[20] Souza B.W., Cerqueira M.A., Bourbon A.I., Pinheiro A.C., Martins J.T., Teixeira J.A., Coimbra M.A., Vicente A.A. (2012). Chemical characterization and antioxidant activity of sulfated polysaccharide from the red seaweed *Gracilaria birdiae*. Food Hydrocol..

[21] Floegel A., Kim D.O., Chung S.J., Koo S.I., Chun O.K. (2011). Comparison of ABTS/DPPH assays to measure antioxidant capacity in popular antioxidant-rich US foods. J. Food Compd. Anal..

[22] Wang J., Zhang Q.B., Zhang Z.S., Li Z.E. (2008). Antioxidant activity of sulfated polysaccharide fractions extracted from *Laminaria japonica*. Int. J. Biol. Macromol..

[23] Wang J.Q., Zhang B., Zhang Z.S., Song H.F., Li P.C. (2010). Potential antioxidant and anticoagulant capacity of low molecular weight fucoidan fractions extracted from *Laminaria japonica*. Int. J. Biol. Macromol..

[24] Qin Y.J., Yuan Q.X., Zhang Y.X., Li J.L., Zhu X.J., Zhao L.L., Wen J., Liu J.K., Zhao L.Y., Zhao J.H. (2018). Enzyme-Assisted Extraction Optimization, Characterization and Antioxidant Activity of Polysaccharides from Sea Cucumber *Phyllophorus proteus*. Molecules.

[25] Di T., Chen G.J., Sun Y., Ou S., Zeng X.X., Ye H. (2017). Antioxidant and immunostimulating activities in vitro of sulfated polysaccharides isolated from *Gracilaria rubra*. J. Funct. Foods.

[26] Shim W.B., Kim K.Y., Chung D.H. (2009). Development and Validation of a Gold Nanoparticle Immunochromatographic Assay (ICG) for the Detection of Zearalenone. J. Agric. Food Chem..

[27] Xu X.F., Yang J.G., Luo Z., Zhang X.W. (2015). Lentinula edodes-derived polysaccharide enhances systemic and mucosal immunity by spatial modulation of intestinal gene expression in mice. Food Funct..

[28] Yuan Q.X., Xie Y.F., Wang W., Yan Y.H., Ye H., Jabbar S., Zeng X.X. (2015). Extraction optimization, characterization and antioxidant activity in vitro of polysaccharides from mulberry (*Morus alba* L.) leaves. Carbohydr. Polym..

[29] Dubois M., Gilles K.A., Hamilton J.K., Rebers P.A., Smith F. (1956). Colorimetric method for determination of sugars and related substances. Anal. Chem..

[30] Bradford M.M. (1976). A rapid and sensitive method for the quantitation of microgram quantities of protein utilizing the principle of protein-dye binding. Anal. Biochem..

[31] Li J.E., Nie S.P., Xie M.Y., Li C. (2014). Isolation and partial characterizationof a neutral polysaccharide from *Mosla chinensis Maxim. cv*. Jiangxiangru and its antioxidant and immunomodulatory activities. J. Funct. Foods.

[32] Blumenkrantz N., Asboe-Hansen G. (1973). New method for quantitative determination of uronic acids. Anal. Biochem..

[33] Doigson K.S., Price R.G. (1962). A note on the determination of the ester sulfate content of sulfated polysaccharides. Biochem. J..

[34] Xie M.H., Hu B., Wang Y., Zeng X.X. (2014). Grafting of gallic acid onto chitosan enhances antioxidant activities and alters rheological properties of the copolymer. J. Agric. Food Chem..

[35] Xiao J.B., Huo J.L., Jiang H.X., Yang F. (2011). Chemical compositions and bioactivites of crude polysaccharides from tea leaves beyond their useful date. Int. J. Biol. Macromol..

[36] Cao J.G., Xia X., Chen X.F., Xiao J.B., Wang Q.X. (2013). Charactetization of flavonoids from *Dryopteris erythrosora* and evaluation of their antioxidant, anticancer and acetylcholinesterase inhibition activities. Food Chem. Toxicol..

[37] Zhu K., Zhou H., Qian H. (2006). Antioxidant and free radical-scavenging activities of wheat germ zsprotein hydrolysates (WGPH) prepared with alcalase. Process Biochem..

